# Eyes as Gateways for Environmental Light to the Substantia Nigra: Relevance in Parkinson's Disease

**DOI:** 10.1155/2014/317879

**Published:** 2014-01-22

**Authors:** Stefania Romeo, Daniela Di Camillo, Alessandra Splendiani, Marta Capannolo, Cristina Rocchi, Gabriella Aloisi, Irene Fasciani, Giovanni U. Corsini, Eugenio Scarnati, Luca Lozzi, Roberto Maggio

**Affiliations:** ^1^Biotechnological and Applied Clinical Sciences Department, University of L'Aquila, 67100 L'Aquila, Italy; ^2^Department of Physical and Chemical Sciences, University of L'Aquila, 67100 L'Aquila, Italy; ^3^Department of Translational Research and New Technology in Medicine, University of Pisa, 56126 Pisa, Italy

## Abstract

Recent data indicates that prolonged bright light exposure of rats induces production of neuromelanin and reduction of tyrosine hydroxylase positive neurons in the *substantia nigra*. This effect was the result of direct light reaching the *substantia nigra* and not due to alteration of circadian rhythms. Here, we measured the spectrum of light reaching the *substantia nigra* in rats and analysed the pathway that light may take to reach this deep brain structure in humans. Wavelength range and light intensity, emitted from a fluorescent tube, were measured, using a stereotaxically implanted optical fibre in the rat mesencephalon. The hypothetical path of environmental light from the eye to the *substantia nigra* in humans was investigated by computed tomography and magnetic resonance imaging. Light with wavelengths greater than 600 nm reached the rat *substantia nigra*, with a peak at 709 nm. Eyes appear to be the gateway for light to the mesencephalon since covering the eyes with aluminum foil reduced light intensity by half. Using computed tomography and magnetic resonance imaging of a human head, we identified the eye and the superior orbital fissure as possible gateways for environmental light to reach the mesencephalon.

## 1. Introduction

Light penetration in biological tissues is a phenomenon that has been studied for many years due to its therapeutic and diagnostic potential [1, 2]. Several parameters affect the penetration of light in living tissues, such as wavelength, intensity, polarization, and coherence of the light source as well as tissue composition [3, 4]. In particular, light absorption in biological tissues is heavily dependent on wavelength. In the ultraviolet range, absorption increases with shorter wavelength due to protein amount, DNA, and other molecules, such as melanin. Conversely, in the infrared range, absorption increases with wavelength due to the presence of water. Although whole blood is a strong absorber of light, its influence on light transport is moderate because the volume of blood in tissues is low. Minimum absorbance, hence, the best transmission of light in tissues, occurs in the red and near infrared range (between 670 and 910 nm), which is a region known as the “diagnostic and therapeutic window” [5].

Biological tissues can detect light through retinal and nonretinal photoreceptors. Nonretinal photoreceptors are present in a variety of vertebrates, with the exception of mammals [6]. They are normally positioned in the diencephalon, playing an important role in endocrine regulation. For example, Opsin 5 in the paraventricular organ of quail appears to be a deep brain photoreceptive molecule that regulates seasonal reproduction in birds [7].

In addition to photoreceptors, other endogenous and exogenous photosensitive compounds can mediate nonvisual responses, and their photoactivation may potentially lead to tissue damage [8]. Consistent with this, we have recently shown that continuous (three months) bright light exposure of rats induces formation of neuromelanin and reduces tyrosine hydroxylase positive neurons in the *substantia nigra* [9]. This effect was the result of direct light reaching the *substantia nigra* and not due to alteration of circadian rhythm since it was also observed in animals rendered completely blind by bilateral transection of the optic nerve.

In the current study, we measured the wavelength range and intensity of light reaching the *substantia nigra* in anaesthetised rats. Furthermore, we analysed the light spectrum of a series of computer screens in order to assess whether this wavelength range was emitted. Finally, in collaboration with a neuroradiologist, we sought to determine potential preferential pathways for light to reach the *substantia nigra* in humans.

## 2. Materials and Methods

### 2.1. Animals

All experiments were performed in compliance with the Animal Care and Use Committee guidelines and approved by the Italian Ministry of Health (Italian Legislative Decree, Directive n.86/609/CEE, January 27th, 1992 n.116). All efforts were made to minimise the number of animals used and their suffering. The data presented in the current study were obtained from experiments carried out on Sprague-Dawley albino rats that weighed between 300 and 350 g.

### 2.2. Stereotaxic Surgery to Implant an Optical Fibre in the Rat *Substantia Nigra*

**Figure 1:**
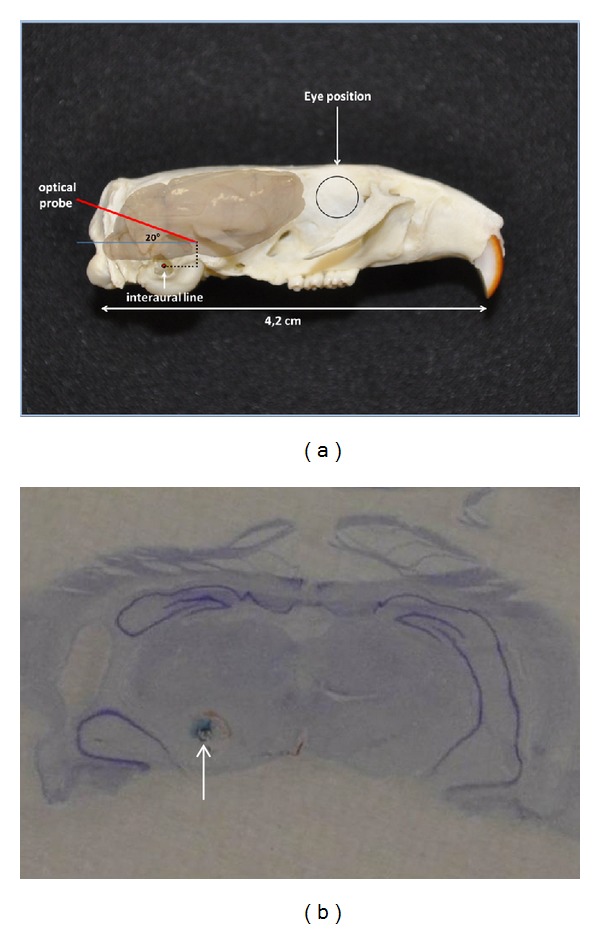
Schematic representation of the transcerebellar trajectory of the optical probe implanted in the rat *substantia nigra*. (a) The final coordinates of the probe tip were AP +4.0, L 2.0, and DV 2.5 from the interaural line, according to the Rat Brain Atlas of Paxinos and Watson. The black circle represents the eye position with respect to the brain. (b) Coronal section (30 *μ*m thick) stained with Cresyl Violet to evaluate the position (arrow) of the optical probe within the mesencephalon.

Animals were anaesthetised with chloral hydrate (400 mg/kg i.p.) and placed in a stereotaxic apparatus (Unimécanique, Epinay-Sur-Seine, France). Given the purpose of the present experiment, it was essential to insert a light measuring probe (optical fibre from Ocean Optics: 600 *μ*m diameter, UV/VIS ~300–1100 nm) in the mesencephalic dopaminergic region. The trajectory of the optical fibre was required to be as horizontal as possible to allow for the maximum incidence of entering light, which would come from the front, top, and lateral of the animal head, upon the exposed transversal surface of the optical probe. To achieve this, the mesencephalic region was targeted with a transcerebellar trajectory inclined 20° on the sagittal plane (Figure 1(a)). Presurgical stereotaxic coordinates with this trajectory were set in the stereotaxic frame in the absence of an animal head. This was carried out using a micromanipulator that held a guide cannula (1.25 mm o.d., 0.90 i.d) whose final tip was positioned 2 mm behind the target point. Thus, the final coordinates for measuring light were AP +4.0, L 2.0, and DV 2.5 from the interaural line, according to the Rat Brain Atlas of Paxinos and Watson. The manipulator was first withdrawn and the animal head was centered and fixed in the stereotactic apparatus. Neck muscles were then excised, the skull exposed, and the manipulator repositioned to the same coordinates after drilling a hole in the interparietal bone, allowing entry of the inclined cannula. Once inserted in the brain, the guide cannula was fixed with acrylic cement, then the light measuring probe was inserted. Light was subsequently measured using a calibrated spectroradiometer (USB2000-XR1 from Ocean Optics).

After acquiring light measurements, the probe was withdrawn and a bipolar coaxial electrode (450 *μ*m O.D., tip-barrel distance 100 *μ*m) was inserted into the guide cannula, protruding 2 mm from the tip of the cannula in exact placement of the original position of the optical probe. A positive current of 10 *μ*A was passed through the filament for 30 sec to mark the site from which the light was measured. Later, under deep chloral hydrate anaesthesia, the rats were sacrificed by perfusion of potassium ferrocyanide. Briefly, animals were intracardially perfused with 50 mL of cold saline containing 0.2 mL of heparin (5000 IU/mL) and 10% potassium ferrocyanide in fixative solution (4% paraformaldehyde). The brain was removed and stored for 48 h in a 3 : 1 solution composed of the perfusion liquid and 95% alcohol with 2% acetic acid. For analysis, the brain was divided into sections using a rodent RBM 400C brain matrix (ASI Instruments, Warren, MI, USA) and then embedded in OCT and frozen. Parallel 30 *μ*m coronal sections, including the mesencephalic region, were cut using a cryostat (Leica) and stained with Cresyl Violet to evaluate the position of the site within the mesencephalon where light measurements were taken (Figure 1(b)).

### 2.3. Neuroradiological Imaging

Three-dimensional brain computed tomography (CT) with 0.5 mm collimation on multidetector row helical CT scanners (Somatom Plus 4 CT scanners; Siemens Medical Systems, Forchheim, Germany) was performed on a patient without clinical signs of Parkinson's disease. The X-ray tube potential and current were approximately 120 kV and 200 mA, respectively. Pitch was 1 and scan revolution time was 0.5 seconds. Three-dimensional reconstructions were performed on workstation using preset volume-rendered and MPR display algorithms.

Magnetic resonance imaging (MRI) of the head of the same individual was performed on a 1.5-Tesla MR scanner using a head coil (General Electric Somatom Plus). In order to manipulate the images and obtain measurements, MPR algorithms obtained by sagittal 3D spin echo T1-weigthed sequence were used.

## 3. Results

### 3.1. Penetration of Light in the Rat *Substantia Nigra*

As mentioned in Section 2, the cannula was implanted in the *substantia nigra* with a transcerebellar trajectory inclined 20° on the sagittal plane in order for the optical probe to detect light coming from the front, top, and lateral of the animal head. Rats still under anaesthesia and positioned in the stereotaxic apparatus were then exposed to fluorescent tube lights (Philips Master TL-D Secura 36W/840 1 SL, 120 cm length), which were placed 30 cm above their head.

**Figure 2:**
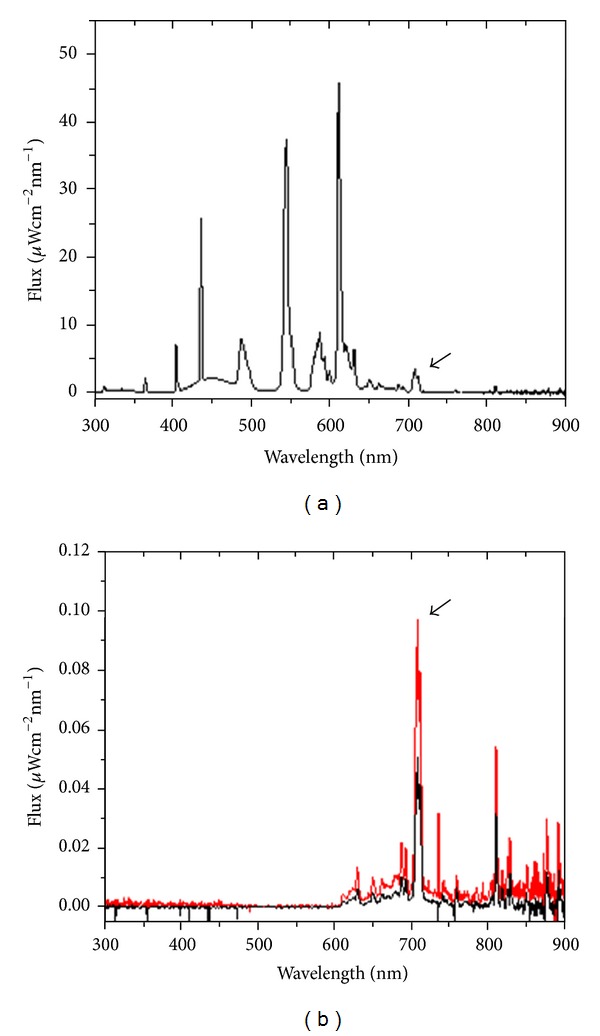
Analysis of the fluorescent light spectrum penetrating into the *substantia nigra*. (a) Spectrum of the fluorescent tube registered by the microoptic fibre directly facing the lamp. (b) Representative light spectrum measured by the optical probe inside the rat brain. The black line and the red line refer to readings taken with covered and uncovered eyes, respectively. The arrows indicate the peaks at ~710 nm.

Figure 2(a) shows the spectrum of the fluorescent tube light that was registered by the microoptical fibre directly facing the lamp. This is the classical profile reported by the manufacturer for this type of fluorescent lamp (http://download.p4c.philips.com/l4b/9/927921484076_eu/927921484076_eu_pss_itait.pdf).

The red line in Figure 2(b) shows a representative spectrum of the light that penetrates the head of the rats and reaches the *substantia nigra*. Clearly, the tissues interposed between the *substantia nigra* and the fluorescent tubes outside absorbed the light with wavelengths less than 600 nm. In order to identify a preferential pathway for light to reach the *substantia nigra*, we covered parts of the animal's head using aluminum foil. A significant reduction (~50%) of the signal was only observed when the eyes were covered (black line in Figure 2(b)).

### 3.2. Light Spectrum of Computer Screens

**Figure 3:**
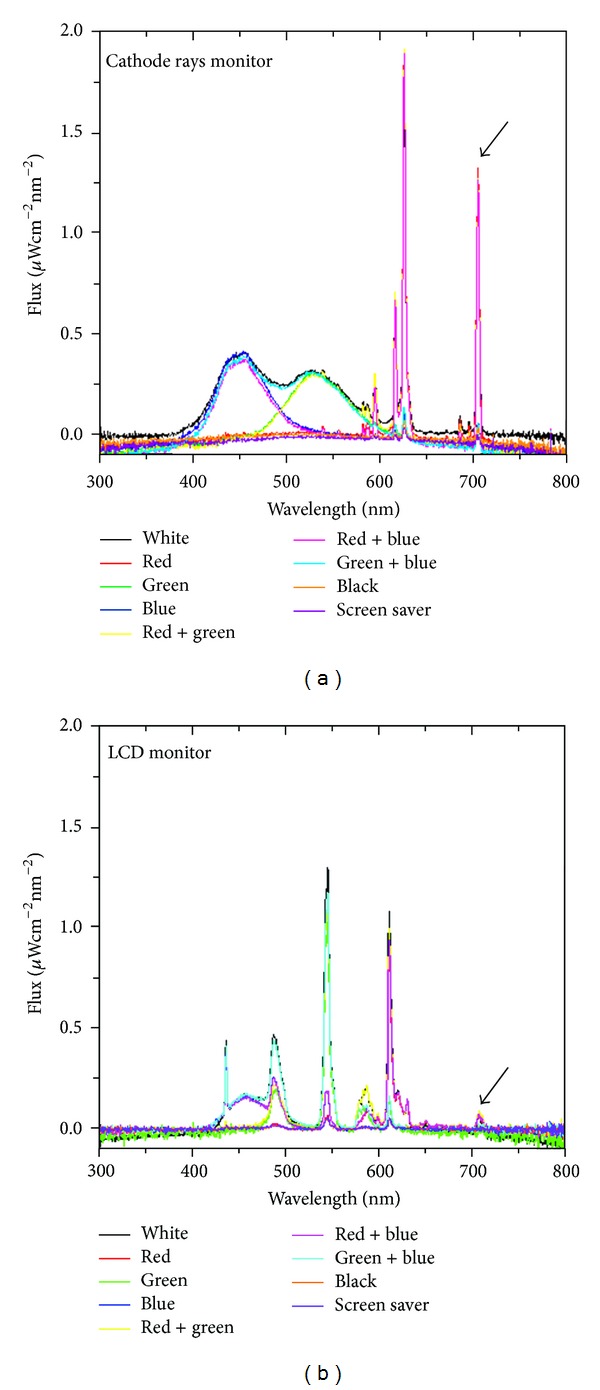
Light spectrum of two different computer monitors. (a) Hitachi CM615 cathode-ray tube (CRT) monitor. (b) ACER AL1916 liquid-crystal display (LCD) monitor. The arrows indicate the peaks at ~710 nm.

Television and computer screens are among the most common light sources that humans are exposed to every day. In order to assess whether these electronic devices emit a wavelength range that may reach the *substantia nigra*, the same calibrated spectroradiometer used for measuring light inside the rat brain was used to analyse the light spectrum of two monitors: a Hitachi CM615 cathode-ray tube (CRT) and an ACER AL1916 liquid-crystal display (LCD) monitor. As shown in Figure 3, within the range of wavelengths capable of reaching the *substantia nigra*, there is a significant difference in the electromagnetic spectrum between the two monitors. The CRT monitor has an emission peak at 706 nm (Figure 3(a)) that was barely visible in the LCD monitor (Figure 3(b)). Even though the total energy emitted by the two monitors was less than the fluorescent lamp, the energy emitted at 706 nm by the CRT monitor (1.3 *μ*W cm^−2^ nm^−1^ or 1.3 *μ*J/s cm^−2^ nm^−1^) was 39% of the spectral irradiance emitted by the fluorescent lamp at 709 nm (3.3 *μ*W cm^−2^ nm^−1^ or 3.3 *μ*J/s cm^−2^ nm^−1^).

### 3.3. Hypothetical Path of Environmental Light to the *Substantia Nigra* in Humans

**Figure 4:**
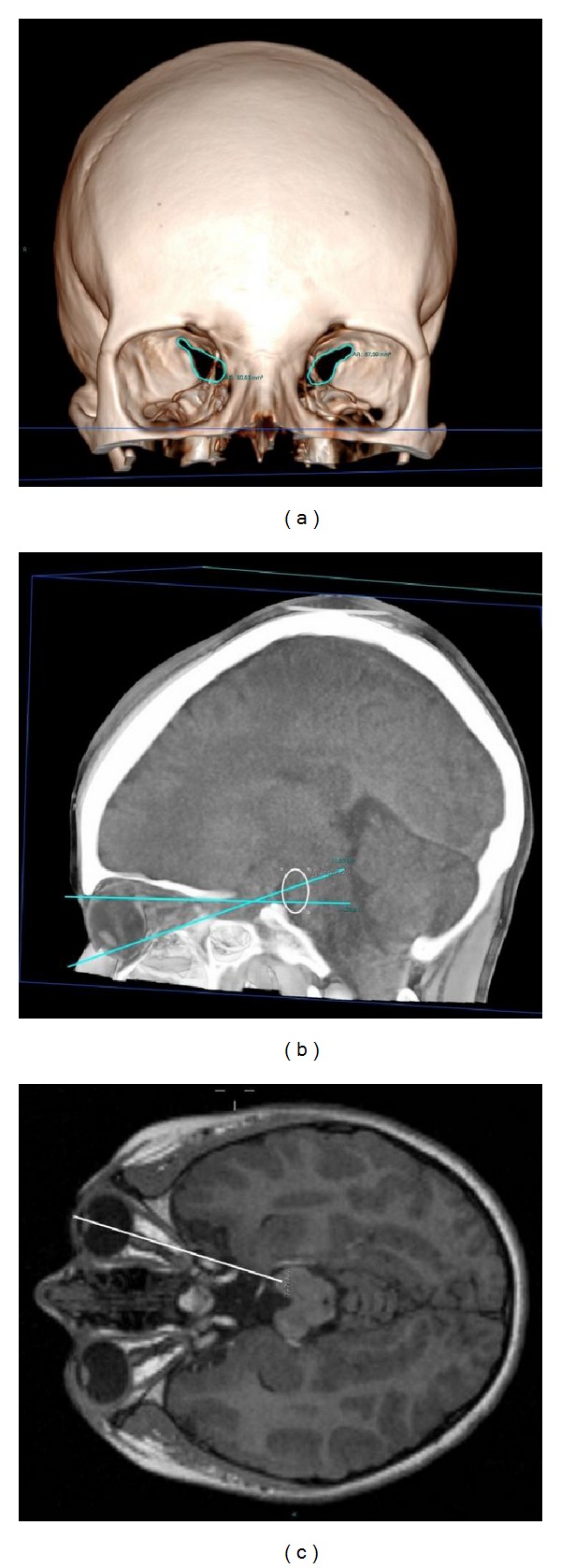
CT and MRI analysis of a human head to trace a possible pathway for light from the outside to the *substantia nigra*. (a) Human head 3D CT scan showing the large superior orbital fissure that gives access to the internal cavity of the skull. (b) Sagittal CT scan of a human head. It is evident from this prospective that the mesencephalon (indicated by the white ellipse) is behind the orbit, not hidden by the posterior clinoid process. (c) Axial MRI scan of a human head. This image shows that CSF occupies a large volume between the superior orbital fissure and the mesencephalon. Furthermore, CSF is present around the optical nerve in the intraconal space. The white line indicates the estimated distance (79.78 mm) between the cornea and the mesencephalon.

We analysed the shortest path that light would have to travel to reach the *substantia nigra* in humans. Analysing the sagittal plane of a human head CT scan (Figure 4(a)), it was evident that light penetrating through the eye to the retina has done already part of its path inside the head. Through the retroorbital tissue, light could then reach the large superior orbital fissure, as shown in the MRI scan in Figure 4(b), gaining access to the internal cavity of the skull. Interestingly, behind the eyeball, in the intraconal space delimited by the four *recti* muscles, there is a layer of cerebrospinal fluid (CSF) between the meningeal sheaths of the optic nerve that extends from the eyeball until the *annulus* of Zinn, from which the *recti* muscles arise. Light could reach the superior orbital fissure crossing part of this fluid path.

From the superior orbital fissure, light could travel through the cerebrospinal fluid behind the lesser wing of the sphenoid and along the edge of the *sella turcica* will go directly into the mesencephalon (Figure 4(a)). The MRI scan in Figure 4(c) clearly shows that liquid occupies a large volume between the superior orbital fissure and the mesencephalon.

In the human subject analysed, this passage between the eye surface and the *substantia nigra* inside the mesencephalon was 79.78 mm long (Figure 4(c)). Within this length, 22.66 mm was comprised of the light transparent tissues of the eye (lens and vitreous humour) and 23.35 mm of liquid behind the superior orbital fissure inside the skull (from the anterior clinoid process to the mesencephalon). As demonstrated, part of the remaining path could be inside the CSF around the optic nerve (9.73 mm). Taken together, the light transparent portion of this path could be as long as 55.74 mm, meaning that at some angle, only 24.04 mm of biological tissue stands between the external light environment and the *substantia nigra* in this patient.

## 4. Discussion

In the current study, we analysed which component of the light spectrum of a fluorescent lamp was able to reach the *substantia nigra* [9]. This was accomplished using a microoptical probe stereotaxically implanted in this deep brain structure. Given the purpose of the experiment, we inserted the light measuring probe into the mesencephalic dopaminergic region with a trajectory as horizontal as possible to allow the maximum incidence of light coming from the frontal, top, and lateral part of the animal head. We observed that only wavelengths above 600 nm reached the *substantia nigra*, with a transmission peak at 709 nm. The tissues interposed between the lamp and the *substantia nigra* attenuated this peak by 34-folds. Nevertheless, it is possible that we underestimated the amount of light reaching this inner structure of the brain because of the slanted angle of the optical probe with respect to the direction of the light penetrating into the brain. This is also suggested by the fact that the amount of light reaching the *substantia nigra* was reduced by increasing the angle of the optical probe with the horizontal plane (data not shown).

Surprisingly, when we shielded the eye ipsilateral to the optical probe, the amount of light reaching the *substantia nigra* was reduced by half, indicating that light may preferentially reach the mesencephalon through the eye. In retrospect, this is intuitive as the eye is transparent to light and it occupies part of the head.

This observation prompted us to search for a preferential pathway for light from the outside to reach the *substantia nigra* in humans. To do so, neuroradiological analysis of a human head revealed that the shortest path from the outside to the midbrain is through the eye. Specifically, light may pass through the lens and vitreous humour of the eye to the retina, then penetrate the retroorbital tissue to the superior orbital fissure, gaining access to the intracranial cavity. Indeed, behind the superior orbital fissure there is a liquid layer as far as the mesencephalon. Since the CSF is transparent to visible light, attenuation of light transmission is most likely to occur in the retroorbital tissue.

Despite the findings from the current study, we do not know whether light can reach the *substantia nigra* through this path. The only way to confirm the pathway of light would be to measure it in a *postmortem* brain. Nonetheless, if the eyes are the gateway for light from the outside to the *substantia nigra*, people who spend a lot of time in front of light sources, such as computers or TV displays, for work, or leisure purposes, may be at increased risk for developing Parkinson's disease. On the contrary, blind people, who do not spend time in front of light sources, may be at lower risk of developing Parkinson's disease. To our knowledge, such epidemiological studies have not been done. Hopefully, neuroepidemiologists will soon become interested in analysing these relationships. Nevertheless, indirect evidence points to this relationship.

An increased risk of Parkinson's disease has been observed in teaching and health care workers [10–12] and generally in subjects with higher education [13]. Conversely, subjects with occupations presumed to involve high levels of physical activity have a decreased risk of Parkinson's disease [13]. These observations may reflect ascertainment bias, resulting from better access to care or, in case of health care workers, they would also be consistent with the neuroinflammatory hypothesis of Parkinson's disease pathogenesis [14] since workers in these occupations have relatively frequent exposure to infectious agents. However, lack of association with work as a nurse argues against this hypothesis [10]. An alternative hypothesis may be that subjects with higher education, including teachers and health care workers, may generally spend more time in front of light sources, such as computers.

In this regard, even though the global intensity of light emitted by a computer display is relatively low compared to a fluorescent lamp, we observed that the light emitted at 706 nm by a CRT monitor was nearly one-third of the light emitted by a fluorescent lamp. Given that the average person may work or look at a computer display for hours on a daily basis, it is possible that some light could reach the *substantia nigra*. As a matter of fact, computer programmers tend to be diagnosed with Parkinson's disease at a younger age compared to other patients, and risk of diagnosis in patient 50 years old or younger is greater in computer programmers [15].

The peak at 706 nm in the LCD monitor analysed is very small; nonetheless, it should be considered that other LCD monitor, which are manufactured with back fluorescent light, a special light source necessary to produce a visible image, have a higher peak in this wavelength range [16].

eHealthMe—Real World Drug Outcomes (http://www.ehealthme.com/) is a platform to study healthcare data in real world and in real time. Data are retrieved from FDA Medwatch, FDA Safety Information, and the Adverse Event Reporting Program. On October 29th, 2013, it was reported that among 14,447 patients with Parkinson's disease from 1977 to 2013, only 10 (0.07%) experienced blindness (http://www.ehealthme.com/cs/parkinson's+disease/blindness). The global estimate of the number of blind people is 0.58%. In the North American continent, the number of blind people is estimated to be 0.35%, which is similar to Europe (0.3%) [17].

From the same eHealthMe platform, blindness was scored 0.25% in depression, 0.34% in multiple sclerosis, 0.36% in osteoporosis and 0.29% in hypertension, then very close to the percentage of blind people in the North American continent reported by Pascolini and Mariotti 2012 [17] suggesting that data from this platform are reliable. The data retrieved from eHealthMe does not specify the cause of blindness in the 10 Parkinson's disease patients but suggests that there is a negative correlation between these two diseases.

The other epidemiological evidence that indirectly indicates that light exposure may be a risk factor for Parkinson's disease comes from a study that used a Utah genealogic database and a statewide cancer registry to examine the relationship between Parkinson's disease and cancer. This study suggests that melanoma and prostate cancer were the only cancers observed with increased incidence among Parkinson's disease patients [18]. Both melanoma [19, 20] and prostate cancer [21] have been associated with increased exposure to light. Interestingly, countries with the highest level of exposure to nightly artificial light have an 80% increase in prostate cancer incidence [21].

## 5. Conclusions

We have provided evidence that light with wavelengths greater than 600 nm can reach the *substantia nigra* in rats and that the preferential pathway to reach this structure is through the eye. Neuroradiological images seem to indicate that the shortest way for light to reach the *substantia nigra* in humans is also through the eye. At the moment, findings from the current study are only speculative. No examples exist to demonstrate that (a) light can reach the* substantia nigra* and that (b) the eyes are the gateway for light from the outside to this deep brain structure; only direct measurement in a *postmortem* brain may substantiate our claims. Nevertheless, we hope that our observations will encourage epidemiological analyses of the relationship between light exposure and Parkinson's disease, highlighting the role of artificial light sources in this disease. While our study points to a negative effect of bright light exposure, it is important to mention that few studies demonstrated a positive effect of bright light therapy on sleep, mood, and motor function in patients with Parkinson's disease [22].
